# Porcine Skin-Derived Silver Nanoparticles: A Novel Green Synthesis Approach and Molecular Characterization of Their Antimicrobial Potential

**DOI:** 10.3390/ijms27083521

**Published:** 2026-04-15

**Authors:** Kyoung Ran Kim, Bummo Koo, Min Woo Lee, Hyeong-Dong Kim, Jong Ryeul Sohn, Suhng Wook Kim

**Affiliations:** 1 Department of Health and Safety Convergence Science, Graduate School, Korea University, 145 Anam-ro, Seoul 02841, Republic of Korea; krank@korea.ac.kr (K.R.K.); bummo0624@naver.com (B.K.); 2 School of Health and Environmental Science, College of Health Science, Korea University, 145 Anam-ro, Seoul 02841, Republic of Korea; leemw@korea.ac.kr (M.W.L.); hdkimx0286@korea.ac.kr (H.-D.K.); 3 Graduate School of Particulate Matter Specialization, Korea University, 145 Anam-ro, Seoul 02841, Republic of Korea; sjr56@naver.com

**Keywords:** silver nanoparticles, porcine skin extracts, green synthesis, antibiotic resistance, antimicrobial activity, biogenic nanoparticle, antimicrobial resistance

## Abstract

Silver nanoparticles (AgNPs) are widely recognized for their potent antibacterial properties and diverse biomedical applications. While conventional synthesis methods typically rely on chemical-reducing agents that may pose risks to human health and the environment, this study proposes an eco-friendly green synthesis approach utilizing porcine skin extracts. The extracts were prepared through thermal treatment and filtration to serve as a biological reducing agent. Successful synthesis was validated using dynamic light scattering, Fourier transform infrared (FTIR) spectroscopy, UV–Vis spectroscopy, and scanning electron microscopy (SEM). Furthermore, the antimicrobial efficacy of the synthesized AgNPs was evaluated against multidrug-resistant microorganisms, demonstrating significant growth inhibition across various antibiotic-resistant strains. These findings suggest that porcine skin—a readily available bioresource—is a promising precursor for the sustainable production of AgNPs with broad-spectrum antimicrobial potential.

## 1. Introduction

Antibiotics, which are antimicrobial agents capable of inhibiting or eradicating microbial proliferation, are extensively employed not only for the therapeutic management of bacterial infections in both human and animal populations, but also for non-medical applications. Recently, due to climate change and environmental pollution, the prevalence of antibiotic-resistant microorganisms has increased [1]. Strong antibacterial agents are required for the treatment of antibiotic-resistant microorganisms; however, these agents may affect the normal flora of the human body, adversely affecting health and placing physical and economic burdens on patients. Therefore, there is an emerging need for novel and eco-friendly antibacterial agents [2,3,4]. AgNPs are well-known materials. Research on the antibacterial and antifungal properties of AgNPs is currently underway [5,6,7]. Hence, AgNPs have applications in biomedical fields, such as biosensors and antimicrobials [8]. In addition, AgNPs are often coated with other materials to prevent microbial infections in public settings and in daily life.

Currently, there are three main methods for synthesizing AgNPs: physical, chemical, and green synthesis. Physical methods, such as high-energy ball milling [9], inert gas condensation [10], laser pyrolysis [11], and electrospinning [12], are typically used to produce high-purity AgNPs with a narrow size distribution.

Chemical methods for synthesizing AgNPs are versatile and can produce AgNPs with a wide range of properties; however, the chemical-reducing agents used in these methods are detrimental to human health and the environment [13,14,15,16,17,18]. Examples of reducing agents currently used to synthesize AgNPs include gamma rays [19,20,21], hydrazine [22], and sodium borohydride [23]. Gamma radiation can cause lethal damage to genes and proteins [24,25]. Hydrazine is toxic via the oral route, skin contact, and inhalation, and may cause skin burns, serious eye damage, and respiratory irritation. Sodium borohydride can cause burns, blindness, and skin damage upon contact, and can result in soil contamination through water pollution [19,20,23,26,27,28,29,30,31,32,33,34].

To overcome this problem, methods for synthesizing nanoparticles from natural products have been developed. This method is known as green synthesis. Green synthetic methods that use naturally occurring materials as reducing agents are environmentally friendly and have the potential to produce AgNPs with novel properties. This eco-friendly approach involves synthesizing metallic nanoparticles using various natural sources such as starch, leaves, roots, flowers, fruits, honey, bacteria, fungi, algae, and microbial enzymes, and is currently receiving extensive research attention [35,36,37,38].

More recently, sophisticated bio-inspired platforms, such as polydopamine-based systems, have emerged as highly efficient and sustainable templates for the controlled synthesis of metallic nanoparticles, further expanding the scope of green nanotechnology beyond traditional plant extracts [39].

However, while green synthesis of AgNPs has predominantly utilized plant extracts, this study shifts the focus toward waste valorization by utilizing porcine skin—a major byproduct of the livestock and food processing industries. Globally, the accumulation of animal-derived waste presents significant environmental challenges. By repurposing this bioresource, we not only reduce the environmental burden but also convert a low-value byproduct into a high-value functional material [40].

Distinct from conventional plant-based green synthesis, the present study explores the potential of animal-derived bioresources by utilizing porcine skin extracts as an eco-friendly reducing agent. Herein, we report the successful synthesis of porcine skin-derived AgNPs and provide a comprehensive assessment of their antimicrobial efficacy against a broad range of both susceptible and multidrug-resistant pathogens.

## 2. Results and Discussion

### 2.1. Green-Synthesized AgNPs Using Porcine Skin

#### 2.1.1. Chromatic Changes in Porcine Skin Induced by AgNO_3_ Treatment

**Figure 1:**
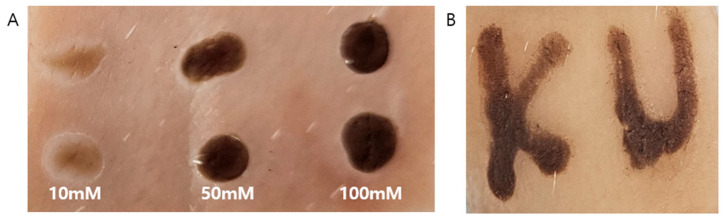
(**A**) AgNO_3_ solutions at concentrations of 10, 50, and 100 mM were reacted with porcine skin for 2 h; (**B**) a 10 mM AgNO_3_ solution was applied to porcine skin and reacted for 24 h.

To observe the chromatic changes of silver nitrate, porcine skin was treated with AgNO_3_ solutions. When 10, 50, and 100 mM AgNO_3_ solutions were applied to pre-washed porcine skin, a distinct color change was observed within 2 h. This coloration intensified in a concentration-dependent manner (Figure 1A). Specifically, the 10 mM treatment resulted in a light brown hue after 2 h, which darkened to a deep brown after 24 h (Figure 1B). These results suggest that endogenous components within the porcine skin reduced the silver ions, leading to the observed pigmentation. These observations indicate the reduction in silver ions and the formation of nanoparticles.

Although the chromatic changes were visually observed, nanoparticle formation was quantitatively monitored using UV-Vis spectroscopy through the evolution of characteristic LSPR peaks.

#### 2.1.2. Preparation of Porcine Skin Extract and Synthesis of AgNPs

**Figure 2:**
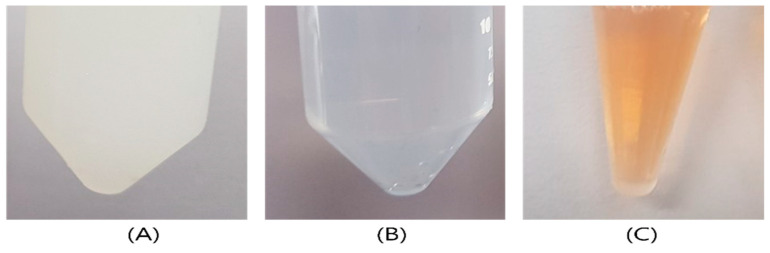
Visual analysis of porcine skin extracts and AgNPs with filtered porcine skin extracts. (**A**) Porcine skin extracts without any filtration. (**B**) Porcine skin extracts filtered twice with a syringe filter (0.2 µm), (**C**) AgNPs synthesized using porcine skin extracts with a syringe filter.

The unfiltered porcine skin extract exhibited high turbidity visible to the naked eye (Figure 2A), whereas the extract filtered through a 0.2 µm syringe filter appeared relatively clear (Figure 2B). The filtered extract was subsequently employed as a reducing agent for the synthesis of AgNPs. The reduction in Ag^+^ ions to Ag^0^ was indicated by a distinct color transition from transparent to brown. When AgNO_3_ was mixed with the extract at a 1:1 ratio, the resulting AgNPs displayed a deep brown color (Figure 2C). The intensity of this coloration is primarily attributed to the Localized Surface Plasmon Resonance (LSPR) effect, which is a characteristic optical property of silver nanoparticles. The environmental sustainability of the proposed synthesis method is rooted in the principle of waste valorization. While the extraction of porcine skin components involves a thermal treatment at 80 °C, this energy expenditure is strategically offset by the elimination of hazardous chemical-reducing agents such as hydrazine and sodium borohydride. Conventional chemical synthesis methods often rely on these substances, which are known to be toxic via skin contact and inhalation, and can lead to significant soil and water contamination. By repurposing porcine skin—a major industrial byproduct—this approach mitigates the environmental burden of biological waste disposal.

### 2.2. Characterization of Synthesized AgNPs Using Porcine Skin Extracts

#### 2.2.1. UV-Vis Analysis of Optical Properties

**Figure 3:**
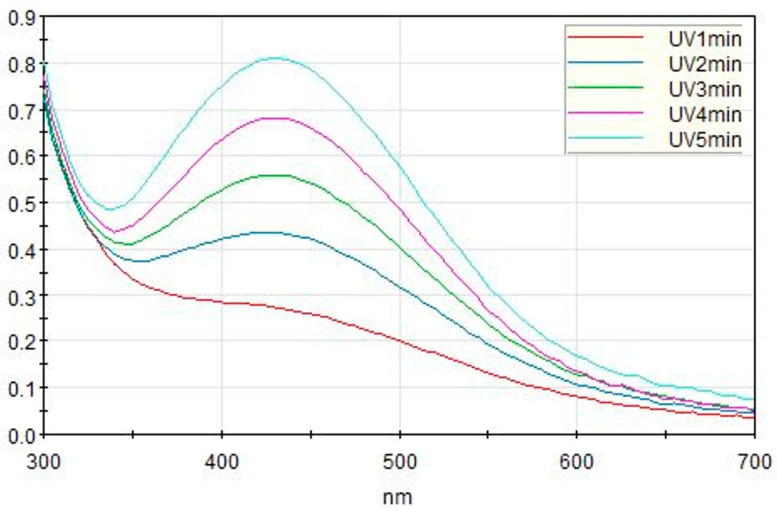
Time-dependent UV-Vis absorption spectra of AgNPs synthesized using porcine skin extracts. The curves show the progression of the reaction from 1 to 5 min, where the emergence and intensification of the peak at ~450 nm confirm the continuous formation and growth of AgNPs.

To monitor the formation of AgNPs in real-time, the UV-Vis spectral changes in the reaction mixture were recorded at 1 min intervals (Figure 3). At the initial stage of the reaction (1 min), the absorbance in the visible range was minimal, indicating the precursor state. However, as the reaction progressed under UV irradiation, a characteristic Localized Surface Plasmon Resonance (LSPR) peak—which is sensitive to nanoparticle size and shape [41]—emerged near 450 nm and its intensity increased rapidly up to 5 min. This spectral evolution provides direct evidence of the successful and progressive bio-reduction in silver ions Ag^+^ into metallic nanoparticles (Ag^0^) mediated by the porcine skin extract.

#### 2.2.2. DLS Analysis of Particle Size Distribution

**Figure 4:**
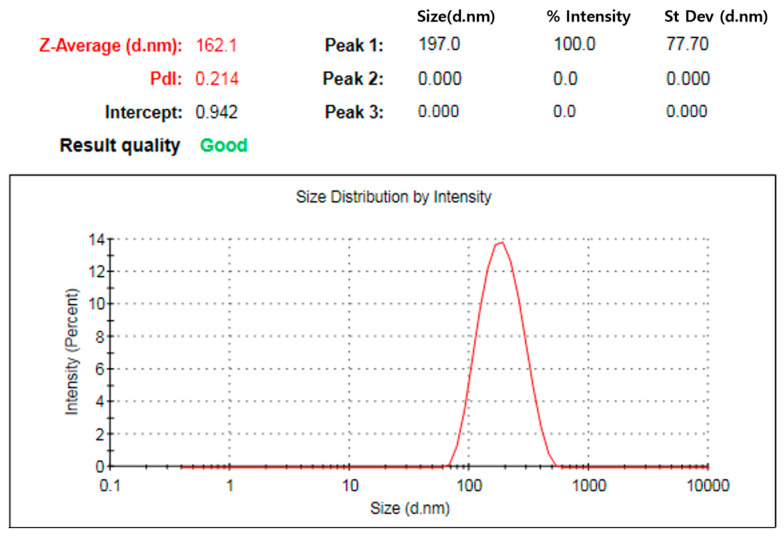
DLS analysis of AgNPs synthesized using porcine skin extracts.

DLS was used to measure the size of the synthesized colloidal particles and confirm their physical, chemical, and biological properties [42]. The synthesized hydrodynamic diameter of AgNPs exhibited low Z-Average (162.1 nm) and PDI (0.214) (Figure 4). The PDI is dimensionless, and the best value obtained from a monodisperse sample is close to 0.05, while the range where the distribution algorithm works best is 0.08 to 0.7 [43]. The synthesized AgNPs exhibited a polydispersity index (PDI) of 0.214, which was lower than 0.7, confirming their superior quality.

#### 2.2.3. SEM Analysis of AgNP Morphology

**Figure 5:**
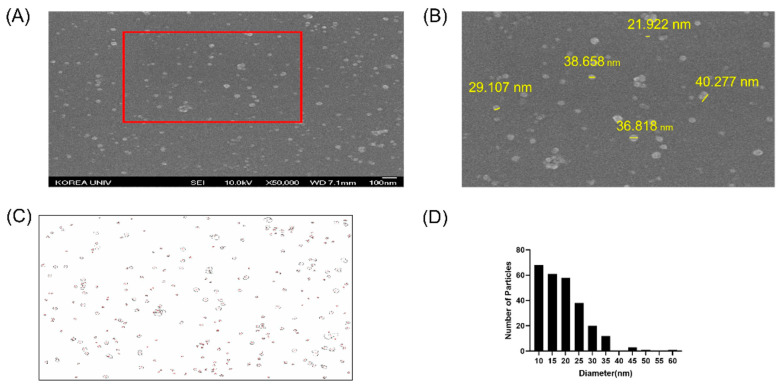
Morphological characterization of AgNPs by SEM. (**A**) AgNPs at 50,000× magnification. (**B**) Particle size of AgNPs via ImageJ calculations. (**C**) Final image employed to measure particle size. (**D**) Size distribution of AgNP diameters.

SEM is typically used to observe the morphology, shape, size, and aggregation of the synthesized nanoparticles [44]. Morphological characterization of the AgNPs was confirmed at 10.0 kV under 50,000× magnification (Figure 5A). SEM revealed that the synthesized AgNPs had a predominantly spherical morphology, with an average particle diameter of 19 nm. The significant discrepancy between the SEM-derived diameter (19 nm) and the DLS-measured Z-average (162.1 nm) is attributed to the fundamental differences in the physical principles of these two characterization techniques. While SEM provides a direct visualization of the dried metallic core, DLS calculates the size based on the Brownian motion of particles in a liquid phase, reflecting the thickness of the protein corona or capping layer formed by the porcine skin-derived biomolecules [45]. Notably, AgNPs did not show any significantly larger aggregates, demonstrating the effectiveness of porcine skin extracts as both a proficient reductant and a capping agent in the synthesis of AgNPs.

#### 2.2.4. Component Analysis of Porcine Skin Extracts via FTIR Spectroscopy

**Figure 6:**
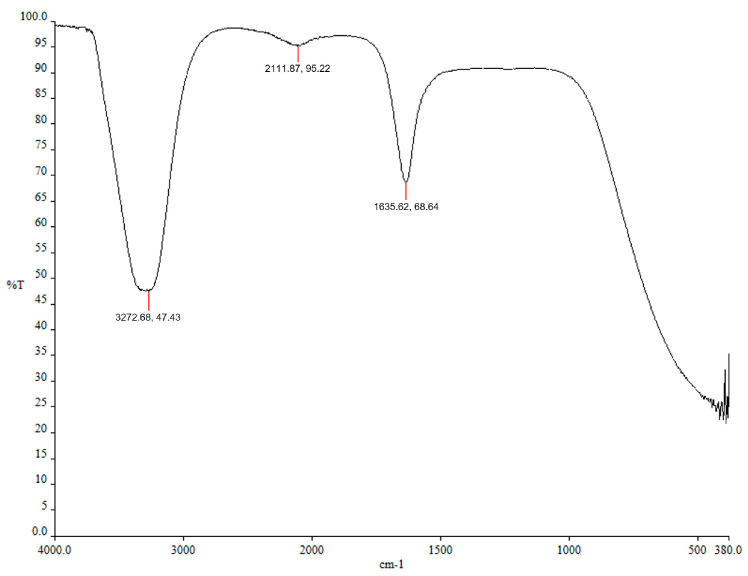
FTIR absorbance peaks of AgNPs synthesized using porcine skin extracts filtered with a syringe filter.

In FTIR analysis (Figure 6), the peak at 1635.62 cm^−1^ corresponds to C=O stretching vibrations of amide and carboxyl functional groups, suggesting the presence of soluble proteins and peptides likely derived from collagen-rich porcine skin tissues during thermal extraction at 80 °C [46]. This interpretation is consistent with previous reports showing that thermal degradation of animal skin collagen yields gelatinous peptides that effectively stabilize metallic nanoparticles [40]. The FTIR spectrum also exhibits a broad absorption band at 3272.68 cm^−1^ corresponding to O–H stretching vibrations of alcohols and phenols, which may also indicate N–H stretching associated with proteinaceous components. The weak peak at 2111.87 cm^−1^ is attributed to the stretching vibration of the C≡C bond in the alkynyl group. These functional groups, including hydroxyl, amide, and carboxyl groups, are known to participate in the reduction in Ag^+^ ions and stabilization of nanoparticles. Therefore, the FTIR results suggest that soluble proteins like collagen present in porcine skin extract may contribute to both the reduction in Ag^+^ ions and the stabilization of nanoparticles during the biosynthesis of AgNPs. These observations support the role of biomolecules derived from porcine skin extract in the green synthesis of AgNPs.

### 2.3. Determination of Biological Activities of Green-Synthesized AgNPs

#### 2.3.1. Antifungal Effect of Green-Synthesized AgNPs on Planktonic *Candida* Species

*Candida* species are normal human microbiota that can colonize human tissues and organs, such as the mouth, throat, gut, and vagina, without causing infection [47]. However, under certain conditions, *Candida albicans* can become a pathogenic fungus, causing infections ranging from serious superficial mucosal infections to life-threatening systemic infections. Therefore, we evaluated the antifungal activity of AgNPs synthesized from porcine skin extracts against *Candida* strains.

**Figure 7:**
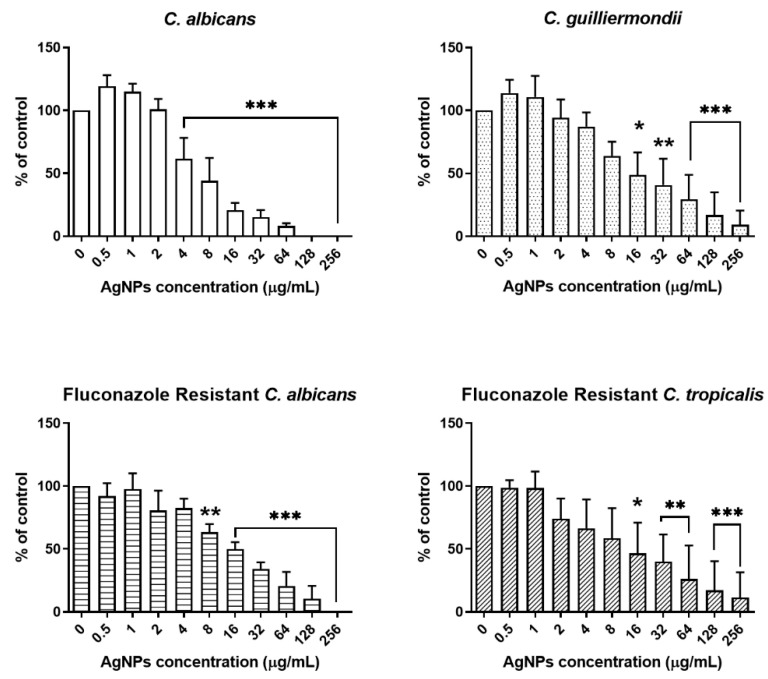
Antifungal activity of AgNPs against *Candida* species. All *Candida* species were treated with AgNPs concentrations ranging from 0.5 to 256 μg/mL. All experiments were performed in triplicate, and statistical significance was determined using one-way ANOVA followed by Dunnett’s post hoc test. * *p* < 0.05, ** *p* < 0.01, and *** *p* < 0.001.

To assess the antifungal effects and MIC_50_ of AgNPs, *Candida* species were treated with AgNPs at concentrations ranging from 0.5 to 256 μg/mL. AgNP concentrations from 4 to 256 μg/mL demonstrated an antifungal effect (Figure 7).

The MIC_50_ of AgNPs against *Candida albicans* was 8 μg/mL. The MIC_50_ for *Candida guilliermondii*, fluconazole-resistant *Candida albicans*, and fluconazole-resistant *Candida tropicalis* was 16 μg/mL. The MIC_50_ range for various *Candida* species was observed to range from 8 to 16 μg/mL, supporting the broad-spectrum antifungal efficacy of AgNPs against both antibiotic-susceptible and antibiotic-resistant fungi, as shown in Table 1.

In addition, no antimicrobial activity was observed in the negative control (deionized water), confirming that the observed inhibitory effects were solely attributable to the synthesized AgNPs.

#### 2.3.2. Antibacterial Effect of Green-Synthesized AgNPs

1. Antibacterial Effect and MIC_50_ of Green-Synthesized AgNPs on Gram-Positive Bacteria

**Figure 8:**
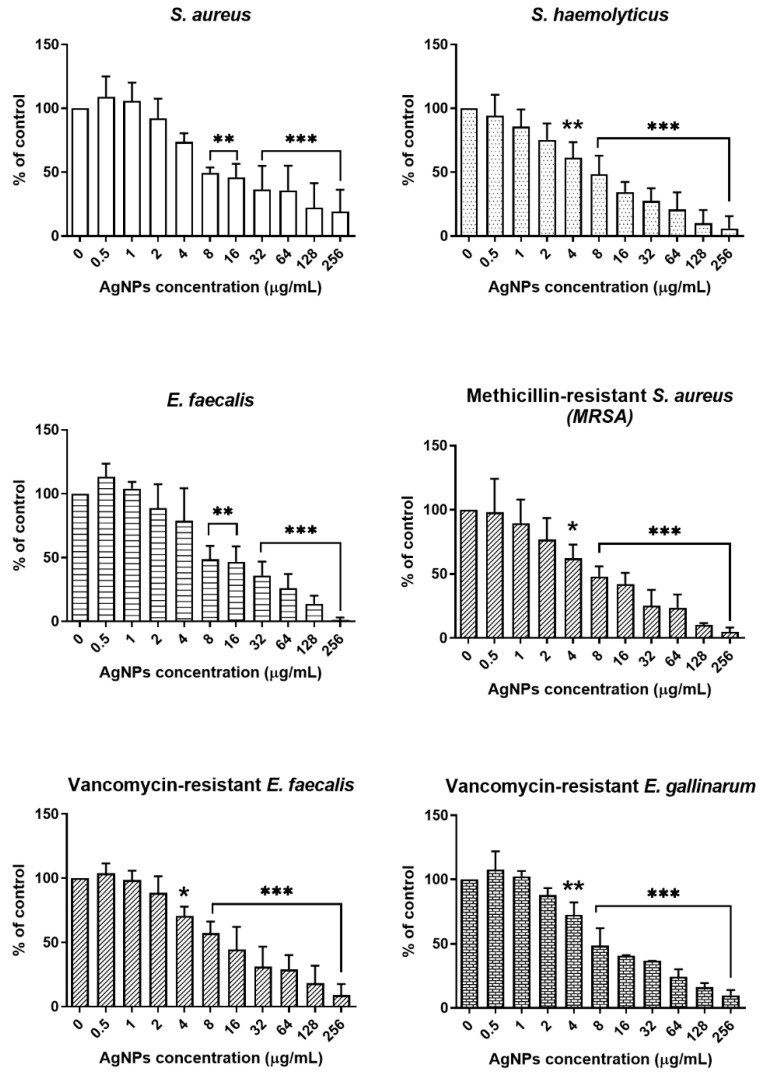
Antibacterial activity of AgNPs against Gram-positive bacteria. All Gram-positive bacterial strains were treated with AgNP concentrations ranging from 0.5 to 256 μg/mL. The data are expressed as mean ± SD (*n* = 3). Statistical analysis was performed using one-way ANOVA with Dunnett’s post hoc test. * *p* < 0.05, ** *p* < 0.01, and *** *p* < 0.001.

The antibacterial activity of AgNPs synthesized from porcine skin extracts was evaluated against six Gram-positive bacterial strains: *Staphylococcus aureus* (*S. aureus*), *Staphylococcus haemolyticus* (*S. haemolyticus*), *Enterococcus faecalis* (*E. faecalis*), Methicillin-resistant *Staphylococcus aureus* (MRSA), vancomycin-resistant *E. faecalis*, and vancomycin-resistant *E. gallinarum*. The bacteria were exposed to AgNP concentrations ranging from 0.5 to 256 μg/mL. AgNPs at concentrations of 4–256 µg/mL exhibited an antibacterial effect against both antibiotic-susceptible and antibiotic-resistant bacteria (Figure 8). The MIC_50_ values for *S. aureus*, *S. haemolyticus*, *E. faecalis*, MRSA, and vancomycin-resistant *E. gallinarum* were all 8 µg/mL. Only the MIC_50_ for vancomycin-resistant *E. faecalis* was confirmed to be 16 µg/mL (Table 2). The potent antimicrobial activity of the synthesized AgNPs against MDR strains, such as MRSA and VRE, is particularly noteworthy. Given that these pathogens exhibit high-level resistance to conventional antibiotics like methicillin and vancomycin, the MIC_50_ values of 8~16 µg/mL obtained in this study demonstrate the potential of porcine skin-derived AgNPs as a robust alternative where standard therapies fail [48].

The bactericidal mechanism of AgNPs is not fully understood; however, it is likely that they act similarly to other antimicrobial agents by disrupting cell wall synthesis, protein synthesis, nucleic acid synthesis, and metabolic pathways [49].

According to research investigating the antibacterial mechanism of AgNPs against *S. aureus*, a representative Gram-positive bacterium, AgNPs damage the structure of the bacterial cell membrane, inhibit cell respiration, and modulate the abundance of certain enzymes [50]. Treatment of *S. aureus* with AgNPs resulted in cell wall breakdown, release of cellular contents, and depletion of the cytoplasm, whereas the untreated *S. aureus* cells retained their smooth surface and typical coccal morphology [50]. These findings provide evidence supporting the potent antibacterial activity of AgNPs.

2. Antibacterial Effect and MIC_50_ of Green-Synthesized AgNPs on Gram-Negative Bacteria

**Figure 9:**
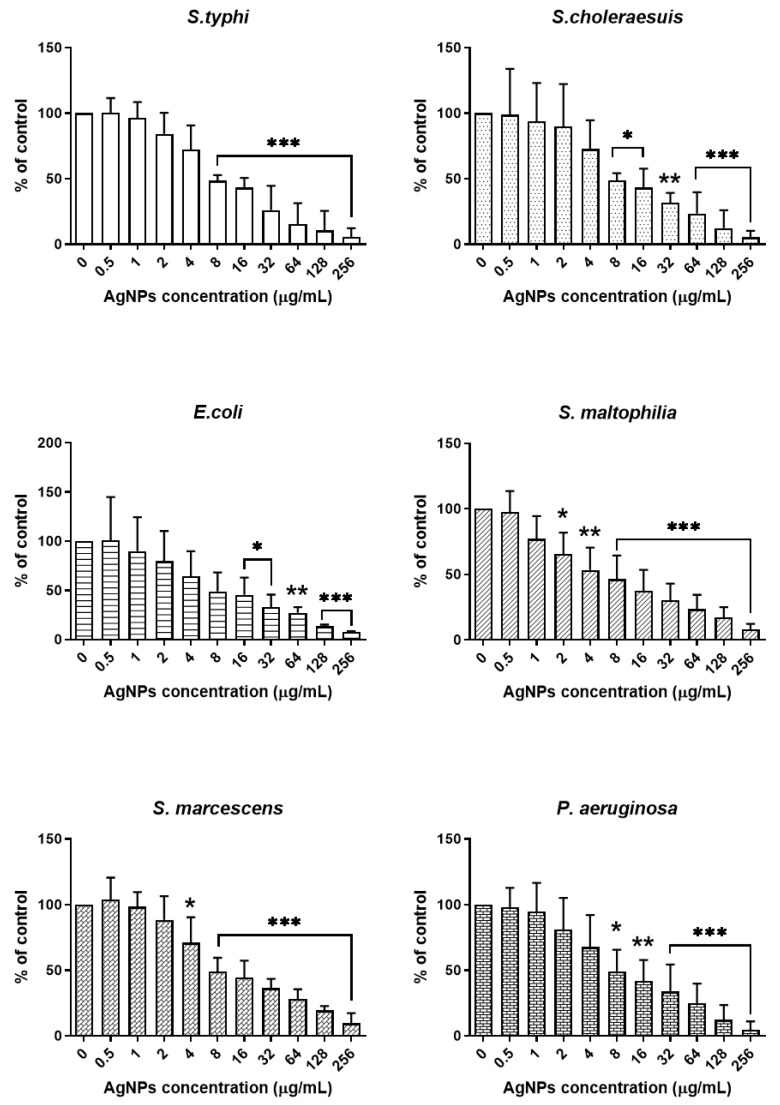

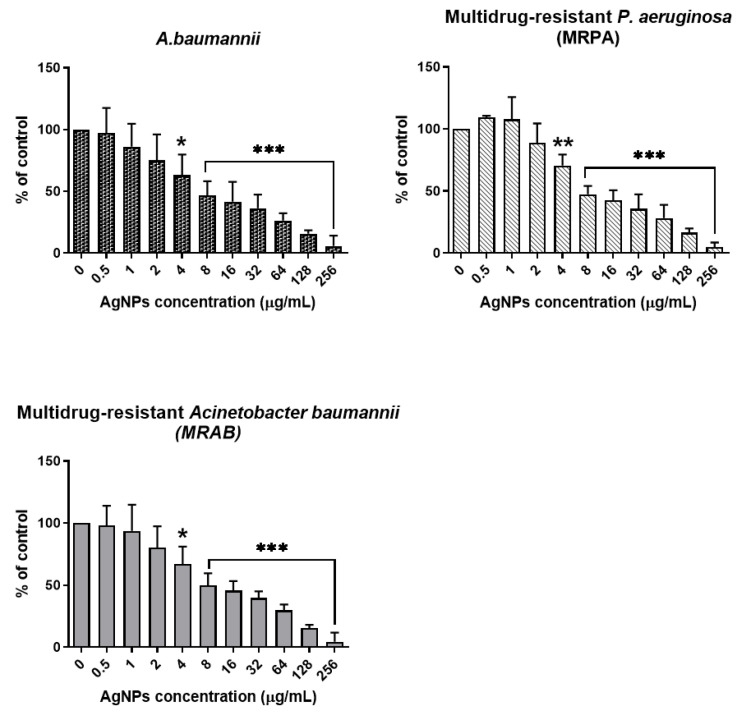
Antibacterial activity of AgNPs against Gram-negative bacteria. All Gram-negative bacterial strains were treated with AgNP concentrations ranging from 0.5 to 256 μg/mL. The data are expressed as mean ± SD (*n* = 3). Statistical analysis was performed using one-way ANOVA with Dunnett’s post hoc test. * *p* < 0.05, ** *p* < 0.01, and *** *p* < 0.001.

Nine strains of Gram-negative bacteria, including *Salmonella enterica serovar* Typhi (*S.* Typhi), *Salmonella enterica serovar* Choleraesuis (*S.* Choleraesuis), *Escherichia coli* (*E. coli*), *Stenotrophomonas maltophilia* (*S. maltophilia*), *Serratia marcescens* (*S. marcescens*), *Pseudomonas aeruginosa* (*P. aeruginosa*), *Acinetobacter baumannii* (*A. baumannii*), Multidrug-resistant *Pseudomonas aeruginosa* (MRPA), and Multidrug-resistant *Acinetobacter baumannii* (MRAB), were used to measure the antibacterial activity of AgNPs synthesized from porcine skin extracts. The bacteria were exposed to AgNP concentrations ranging from 0.5 to 256 μg/mL. AgNPs at concentrations of 2–256 µg/mL exhibited an antibacterial effect against both antibiotic-susceptible and resistant bacteria (Figure 9). The MIC_50_ values for all tested Gram-negative bacteria were 8 µg/mL (Table 3). Thus, the antibacterial effects of AgNPs were observed in both antibiotic-susceptible and antibiotic-resistant Gram-negative bacteria. These findings demonstrate the antibacterial effect of AgNPs synthesized using porcine skin extracts against Gram-negative bacterial strains.

The antimicrobial activity of AgNPs against Gram-negative bacteria, including *E. coli*, a typical Gram-negative bacterium, has been extensively studied. Exposure to antibacterial concentrations of AgNPs resulted in the accumulation of envelope protein precursors in *E. coli* cells, suggesting that AgNPs target the bacterial membrane and dissipate proton motive forces [51]. When AgNPs enter a bacterial cell, they form a low-molecular-weight region inside the bacteria, causing the bacteria to conglomerate and protect their DNA. As a result, the nanoparticles preferentially attack the respiratory chain and induce cell division, ultimately leading to cell death [52].

## 3. Materials and Methods

### 3.1. Preparation of Porcine Skin Extract

Porcine skin was obtained from a local market (Seoul, Republic of Korea) and washed with deionized water (DW). For the color change observation, the skin samples were reacted directly with AgNO_3_ solutions at concentrations of 10, 50, and 100 mM. Separately, to prepare the porcine skin extract, 5 g of untreated porcine skin was minced and mixed with 100 mL of secondary DW. The mixture was then incubated at 80 °C for 30 min using a constant temperature water bath. Subsequently, the mixture was centrifuged (1524 microcentrifuge, LaboGene, Seoul, Republic of Korea) at 8000 rpm for 15 min. The resulting supernatant was collected and filtered twice through 0.2 µm syringe filters (Minisart, Sartorius, Aubagne, France).

### 3.2. Green Synthesis of AgNPs

A 1 mM solution of silver nitrate (AgNO_3_, Sigma-Aldrich, St. Louis, MO, USA) was mixed with the filtered porcine skin extracts at a 1:1 ratio. The mixture was incubated at room temperature with mild agitation for 48 h in the dark. To ensure that the observed biological effects were solely due to the AgNPs and not residual extract components, a rigorous purification process was performed. The synthesized AgNPs were centrifuged at 13,000 rpm for 15 min. The resulting AgNP pellet was washed with deionized water (DW), and this washing process was repeated three times to thoroughly remove any remaining biological molecules from the porcine skin extract. Finally, the purified AgNPs were reconstituted in DW (or freeze-dried to obtain a powder for precise concentration control) and stored at 4 °C for subsequent experimental use. A control solution (Negative Control) was prepared using deionized water or AgNO_3_ solution without the addition of porcine skin extracts to confirm the baseline.

### 3.3. Characterization of Green-Synthesized AgNPs

The absorbance of the AgNPs was measured at 300–700 nm using a UV-Vis spectrophotometer (EVOLUTION60S, Thermo Fisher Scientific, Inc., Waltham, MA, USA). Colloidal stability was assessed by dynamic light scattering (DLS) with a Zetasizer Nano S90 System (Malvern, UK) to measure the particle size distribution [53,54]. The size and morphology of the synthesized AgNPs were analyzed by SEM. SEM images were obtained using a JSM-6701F SEM instrument from JEOL Ltd. (Tokyo, Japan) at 10 kV accelerating voltage. A colloidal AgNP solution was then applied to a cover glass. After fixing the cover glass coated with AgNPs in 2.5% glutaraldehyde overnight, the cover glass was washed with phosphate-buffered saline (PBS) to begin dehydration. Dehydration was performed using a graded series of ethanol (30%, 50%, 70%, 90%, 95%, and 100%) for 15 min each. The samples were dried with ethanol and cover-slipped in a dryer for 24 h. The dried samples were coated with platinum using an automatic magnetron sputter-coating system (JEOL, Ltd., Japan), and the synthesis of AgNPs was observed using SEM. The AgNPs in the captured images were manually traced and quantified using the public-domain software ImageJ (version 1.54, National Institutes of Health, Bethesda, MD, USA) and its specific tools.

FTIR spectroscopy was used to identify functional groups and chemical bonds associated with the porcine skin extract and the surface capping agents on the synthesized AgNPs. The AgNPs synthesized using porcine skin extracts were filtered through a syringe filter and analyzed using an FTIR spectrometer (Spectrum 100, PerkinElmer, Norwalk, CT, USA) over the range of 4000–400 cm^−1^.

### 3.4. Analysis of the Antifungal Effect of Green-Synthesized AgNPs

#### 3.4.1. Incubation of Fungal Species

Four strains of *Candida* species were cultured in yeast extract peptone dextrose (YPD) medium at 37 °C for 24 h. After cultivation, cells were harvested by centrifugation at 5000 rpm for 5 min, and the supernatant was discarded. The cell pellet was washed twice with 1× PBS and resuspended in RPMI 1640 medium (Sigma-Aldrich). The cell concentration was adjusted to 1 × 10^6^ cells/mL.

#### 3.4.2. Antifungal Activity Analysis of AgNPs Against *Candida* Species

The concentration of AgNPs was adjusted in a range from 0.5 to 256 μg/mL through a two-fold serial dilution method, and subsequently dispensed into 96-well flat-bottomed microtiter plates. In total, 98 μL of the adjusted *Candida* species solution (Table 4) and 2 μL of AgNPs were added to a 96-well plate. The 96-well plates were incubated at 37 °C for 24 h. After incubation, the cells in each well were resuspended by pipetting to remove the effect of the precipitated cells on absorbance, which was measured at 630 nm using a microplate reader (ChroMate Microplate Reader, Awareness Technology, Palm City, FL, USA). The MIC_50_ was defined as the lowest culture concentration at which the absorbance decreased below 50% of the control.

### 3.5. Analysis of the Antimicrobial Effect of Green-Synthesized AgNPs

#### 3.5.1. Incubation of Bacteria

The bacteria were divided into two groups: Gram-positive and Gram-negative. Six strains of Gram-positive bacteria and nine strains of Gram-negative bacteria were selected to evaluate the antibacterial activity of the AgNPs, as listed in Table 5. The selected Gram-positive and Gram-negative bacteria were cultured in Luria–Bertani (LB) broth (Difco Laboratories, Detroit, MI, USA) at 37 °C for 24 h. After incubation, the culture solution was washed twice with 1× PBS. The concentration of bacterial cells was adjusted to 0.5 McFarland (1–2 × 10^8^ CFU/mL) using Mueller–Hinton broth (Difco Laboratories, Detroit, MI, USA).

#### 3.5.2. Antibacterial Activity Analysis of AgNPs Against Bacterial Strains

The concentration of AgNPs was adjusted in a range from 0.5 to 256 μg/mL through a two-fold serial dilution method, and subsequently dispensed into 96-well flat-bottomed microtiter plates. In total, 188 μL of Mueller–Hinton broth, 10 μL of bacterial cell suspension, and 2 μL of AgNPs were added to a 96-well plate. The 96-well plates were incubated at 37 °C for 24 h. After incubation, the cells in each well were resuspended by pipetting to remove the effect of the precipitated cells on absorbance, which was measured at 630 nm using a microplate reader. The MIC_50_ was defined as the lowest culture concentration at which the absorbance decreased below 50% of the control.

### 3.6. Statistical Analysis

Each experiment was performed in triplicate (*n* = 3). All results are presented as the means ± standard deviation (SD). Statistical analysis was performed using one-way analysis of variance (ANOVA) with Dunnett’s multiple comparison test using GraphPad Prism 9.5.1 software (Boston, MA, USA). In all analyses, *p* < 0.05 was considered statistically significant, * *p* < 0.05, ** *p* < 0.01, and *** *p* < 0.001, compared to the control group.

## 4. Conclusions

In the present study, AgNPs were successfully synthesized using an eco-friendly green approach with porcine skin extracts as a reducing agent. The synthesized AgNPs exhibited a predominantly spherical morphology with an average diameter of 19 nm and demonstrated potent, broad-spectrum antimicrobial activity against both susceptible and multidrug-resistant pathogens, including MRSA, VRE, and *Candida* species (MIC_50_: 8–16 μg/mL). These findings highlight that porcine skin—a readily available bioresource—can be effectively repurposed to produce high-quality AgNPs, offering a sustainable and cost-effective strategy for utilizing animal by-products to address the global challenge of antibiotic resistance. To minimize variability, standardized extraction conditions were applied; however, further studies are required to assess batch-to-batch reproducibility and scalability under different biological source conditions. While the present findings support the immediate functional stability of the synthesized AgNPs under experimental conditions, further investigations are necessary to evaluate their long-term stability (shelf-life) and biocompatibility in complex biological systems. Future research will focus on detailed cytotoxicity assessments and comprehensive physicochemical characterization—including X-ray diffraction (XRD) and energy-dispersive X-ray spectroscopy (EDS) to confirm crystallinity and elemental composition—to ensure the safety of these nanoparticles for clinical and environmental applications. Overall, this waste-to-resource strategy provides a robust foundation for sustainable nanotechnology and the development of effective antimicrobial agents.

## Figures and Tables

**Table 1:** MIC_50_ of AgNPs against *Candida* species.

Antibiotic (Fluconazole)	Fungus	MIC_50_
Susceptible	*Candida albicans*	8 µg/mL
	*Candida guilliermondii*	16 µg/mL
Resistant	*Candida albicans*	16 µg/mL
	*Candida tropicalis*	16 µg/mL

**Table 2:** MIC_50_ of AgNPs against Gram-positive bacteria.

Antibiotic	Bacterial Strains	MIC_50_
Susceptible	*Staphylococcus aureus*	8 µg/mL
	*Staphylococcus haemolyticus*	8 µg/mL
	*Enterococcus faecalis*	8 µg/mL
Resistant	Vancomycin-resistant *Enterococcus faecalis*	16 µg/mL
	Vancomycin-resistant *Enterococcus gallinarum*	8 µg/mL
	Methicillin-resistant *Staphylococcus aureus* (MRSA)	8 µg/mL

**Table 3:** MIC_50_ of AgNPs against Gram-negative bacteria.

Antibiotic	Bacterial Strains	MIC_50_
Susceptible	*Salmonella enterica serovar* Typhi	8 µg/mL
	*Salmonella enterica serovar* Choleraesuis	8 µg/mL
	*Escherichia coli*	8 µg/mL
	*Stenotrophomonas maltophilia*	8 µg/mL
	*Serratia marcescens*	8 µg/mL
	*Pseudomonas aeruginosa*	8 µg/mL
	*Acinetobacter baumannii*	8 µg/mL
Resistant	Multidrug-resistant *Pseudomonas aeruginosa* (MRPA)	8 µg/mL
	Multidrug-resistant *Acinetobacter baumannii* (MRAB)	8 µg/mL

**Table 4:** *Candida* species used in the antifungal assay.

Antibiotic	Fungus	Strain
Fluconazole-susceptible fungi	*Candida albicans* (*C. albicans*)	ATCC 90028
	*Candida guilliermondii* (*C. guilliermondii*)	KCMF 20104
Fluconazole-resistant fungi	*Candida albicans* (*C. albicans*)	KCMF 20017
	*Candida tropicalis* (*C. tropicalis*)	KCMF 20197

**Table 5:** Bacterial strains used in the antibacterial assay.

	Bacterium	Strain
Gram-positive bacteria	*Staphylococcus aureus* (*S. aureus*)	KCCM 40881
	*Staphylococcus haemolyticus* (*S. haemolyticus*)	KCCM 42267
	*Enterococcus faecalis* (*E. faecalis*)	KCTC 3206
	Vancomycin-resistant *Enterococcus faecalis* (VRE *E. faecalis*)	CCARM 5025
	Vancomycin-resistant *Enterococcus gallinarum* (VRE *E. gallinarum*)	CCARM 5026
	Methicillin-resistant *Staphylococcus aureus* (MRSA)	CCARM 3089
Gram-negative bacteria	*Salmonella enterica serovar* Typhi (*S.* Typhi)	ATCC 700931
	*Salmonella enterica serovar* Choleraesuis (*S.* Choleraesuis)	ATCC 13312
	*Escherichia coli* (*E. coli*)	KCCM 11234
	*Stenotrophomonas maltophilia* (*S.* *maltophilia*)	KCCM 40270
	*Serratia marcescens* (*S. marcescens*)	KCCM 11809
	*Pseudomonas aeruginosa* (*P. aeruginosa*)	KCTC 1637
	*Acinetobacter baumannii* (*A. baumannii*)	KCTC 2508
	Multidrug-resistant *Pseudomonas aeruginosa* (MRPA)	CCARM 2092
	Multidrug-resistant *Acinetobacter baumannii* (MRAB)	CCARM 12005

## Data Availability

The original contributions presented in this study are included in the article. Further inquiries can be directed to the corresponding author.
